# Supplementary material for: Effects of Fe Impurities on Self-Discharge Performance of Carbon-Based Supercapacitors
Source: Materials (Basel). 2021 Apr 11;14(8):1908. doi: 10.3390/ma14081908 (PMC8070237; doi:10.3390/ma14081908)
Supplement: Supplementary file 1 [file materials-14-01908-s001.pdf]

materials

MDPI

Supporting Materials

Effects of Fe Impurities on Self-Discharge Performance of Carbon-Based Supercapacitors

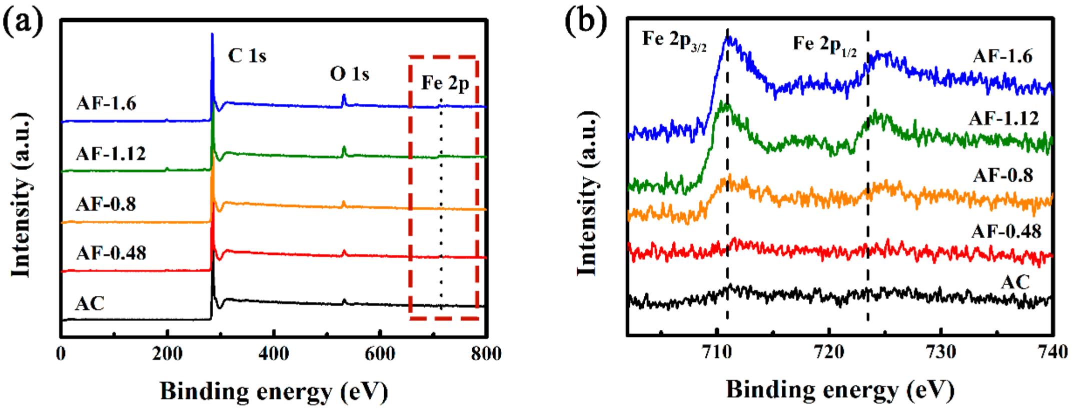

Figure S1. X-ray photoelectron wide scan spectra (**a**) and Fe 2p spectra (**b**) of AC and the Fe@C composites.

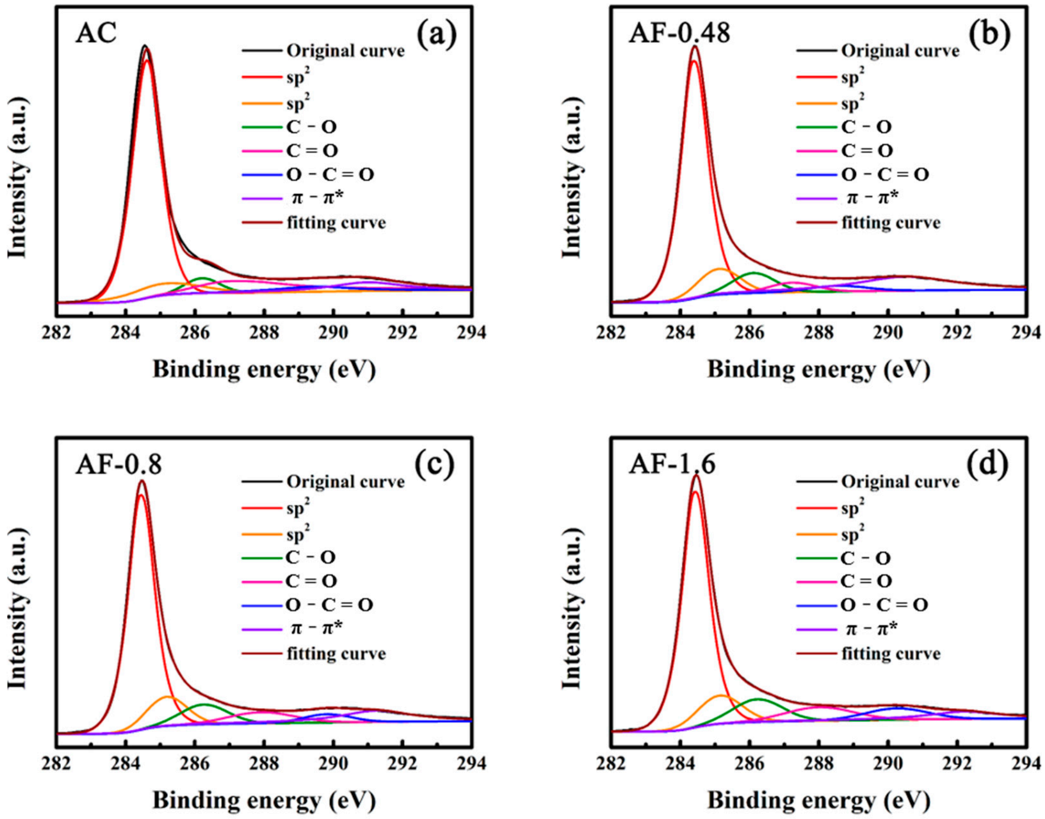

Figure S2. C1s spectra of AC (**a**), AF-0.48 (**b**), AF-0.8 (**c**) and AF-1.6 (**d**).

Table S1. The amounts of Fe in AC and Fe@C composites are investigated by ICP-MS.

Samples	AC	AF-0.48	AF-0.8	AF-1.12	AF-1.6
Fe (mg kg^{-1})	425.0	3260.2	4625.3	6952.0	9911.5

Table S2. Atomic percentage concentrations (%) of the peak area at C, O and Fe binding energy in the XPS spectra (a) and Percentage (%) of the peak area at sp^2 and sp^3 binding energy in the XPS spectra of C1s.

Samples	C (a %)	O (a %)	Fe (a %)	sp^2 (sp2/C a%)	sp^3 (sp2/C a%)
AC	97.17	2.74	0.09	63.87	7.67
AF-0.48	96.61	3.19	0.2	61.2	9.31
AF-0.8	95.95	3.65	0.4	61.1	10.46
AF-1.12	93.26	6.14	0.6	60.1	10.59
AF-1.6	92.6	6.7	0.7	59.53	10.63

Table S3. Specific surface areas of AC and Fe@C composites are investigated by Ar adsorption isotherms at 87 K.

Samples	AC	AF-0.48	AF-0.8	AF-1.12	AF-1.6
Specific surface area (m^2 g^{-1})	1503.9	1676.81	1621.04	1518.52	1586.46

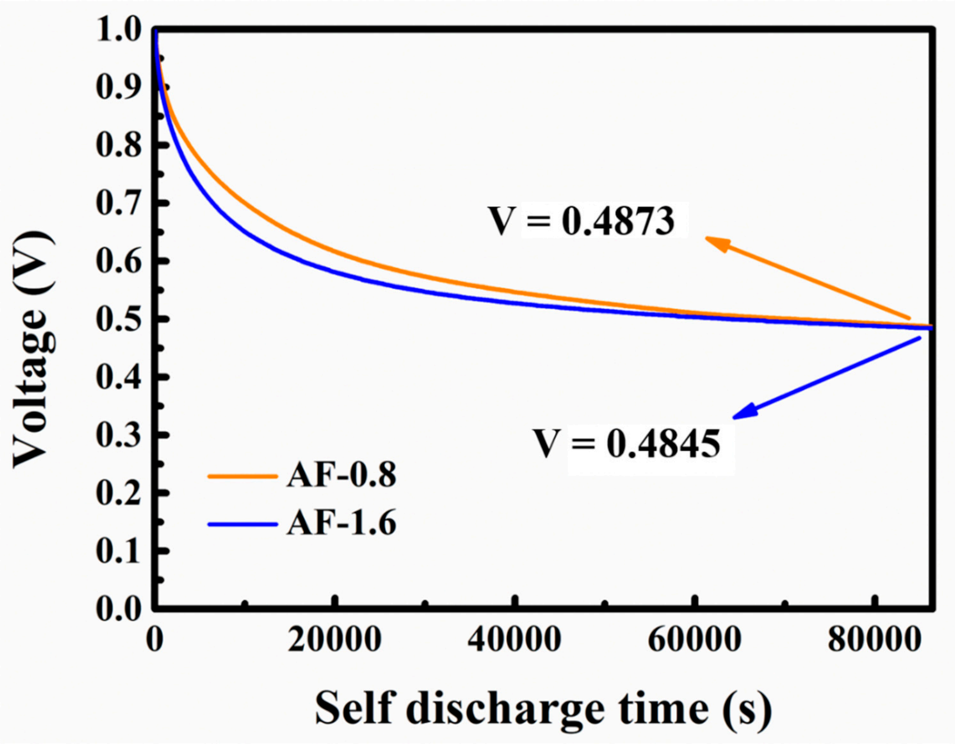

Figure S3. Self-discharge curves of AF-0.8 and AF-1.6.
